# Evaluation of face shields used during aerosol generating procedures

**DOI:** 10.1038/s41598-023-42403-8

**Published:** 2023-09-20

**Authors:** Alessandra A. Pratt, Grant D. Brown, Jarrett E. Walsh, Henry T. Hoffman, Matthew W. Nonnenmann

**Affiliations:** 1 Department of Occupational and Environmental Health, University of Iowa, 145 N Riverside Dr., Iowa City, IA 52242 USA; 2 Iowa City VA Health Care System (152), 601 Highway 6 West, Building 42, Iowa City, IA 52242 USA; 3 Department of Biostatistics, University of Iowa, 145 N Riverside Dr., Iowa City, IA 52242 USA; 4 Department of Otolaryngology, Carver College of Medicine, University of Iowa, 375 Newton Road, Iowa City, IA 52242 USA; 5 Department of Environmental, Agricultural and Occupational Health, University of Nebraska Medical Center, 42nd and Emilie, Omaha, NE 68198 USA

**Keywords:** Epidemiology, Policy and public health in microbiology

## Abstract

Transnasal flexible laryngoscopy is considered an aerosol generating procedure. A negative pressure face shield (NPFS) was developed to control aerosol from the patient during laryngoscopy. The purpose of this study was to determine the effectiveness of the NPFS at controlling virus aerosol compared to a standard disposable plastic face shield. The face shields were placed on a simulated patient coughing machine. MS2 bacteriophage was used as a surrogate for SARS-CoV-2 and was aerosolized using the coughing machine. The aerosolized virus was sampled on the inside and outside of the face shields. The virus aerosol concentration was not significantly different between the inside and outside of the traditional plastic face shield (p = 0.12). However, the particle concentrations across all particle sizes measured were significantly decreased outside the face shield. The virus and particle concentrations were significantly decreased (p < 0.01) outside the NPFS operating at a flow rate of 38.6 L per minute (LPM). When the NPFS was operated at 10 LPM, virus concentrations were not significantly different (p = 0.09) across the face shield. However, the number particle concentrations across all particle sizes measured were significantly different (p < 0.05).

## Introduction

Health care workers (HCWs) experience inhalation exposure to viruses while performing aerosol generating procedures^1,2^. Aerosol generating-procedures are medical procedures that can increase transmission risk for respiratory pathogens because they generate aerosols^1,2^. There is some debate over which specific procedures are considered aerosol generating however, there is a consensus that special care should be taken by the HCW when performing the procedures involving the upper airway in order to ensure adequate respiratory protection^2^. High risk aerosol generating procedure include endotracheal intubation and extubation, high frequency oscillatory ventilation, cardio-pulmonary resuscitation (CPR), bronchoscopy and bronchoalveolar lavage, laryngoscopy, nasopharyngeal washing, aspirate and scoping, and sputum induction^3^. During the pandemic, some elective surgeries have been rescheduled or cancelled due to overflowing capacity in the hospital (lack of hospital beds)^4^. During the COVID-19 pandemic, the use of transnasal laryngoscopy was markedly attenuated due to concern about inducing bioaerosol spread—particularly by inducing a cough. HCWs are exposed to virus aerosols and other pathogens from the equipment used during the procedure as well as when a patient coughs which typically occurs when the surgical equipment is removed from the nose or mouth^5^. HCWs in otolaryngology clinics have been found to be at a higher risk of exposure to SARS-CoV-2 than other specialties and therefore special care should be taken to control their exposure^6^.

A commonly used control measure in otolaryngology clinics during these aerosol generating procedures is personal protective equipment (PPE)^6^. PPE is considered not as effective of an exposure control measure because the HCW is required to correctly wear the protection device and they are still exposed to the hazard^7^. Therefore, PPE should be used as a last resort to control for a hazard or used in conjunction with another control measure. However, PPE use is required during healthcare since the HCW can be exposed when treating patients with viral related illness. To control for hazards during patient care through the pandemic, engineering controls were analyzed^8^. Ventilation is a type of engineering control where the focus is on the amount of air changes per hour in a room to control exposure to SARS-CoV-2^8^. However, providers still had close contact with patients and PPE was required and recommended^6^.

PPE worn by patients varies by their health status and procedure being done in the hospital^9^. For example, a patient that is undergoing laryngoscopy cannot wear a traditional plastic facemask, which could be modified to be worn during the procedure. These traditional face shields have been reported to be ineffective in eliminating virus aerosols^10^. Modifications could include two holes in the front of the face shield so the provider could still use a laryngoscope in the patient’s nose and/or mouth.

A NPFS was designed to be more protective to the provider than a typical face shield or no PPE at all when worn by a patient during laryngoscopy^11^. The NPFS is a hard acrylic shield that has flanged sides and is supported by a heavy-duty clamp and stand (Matthews Hollywood Century 40″ S Stand for Grip Arm Kit, Adorama, Inc, New York, New York). A suction pump is connected to the side of the NPFS using tubing to evacuate air away from the face at approximately 38.6 LPM (the maximum flow rate of the pump). The NPFS combines PPE and engineering control technology to improve biocontainment. On the front of the NPFS, there are two ports for the scope to access the nose or mouth. The shield is placed in front of the patient with their nose is approximately 5 cm away from the front of the shield. A sterilization wrap (Halyard H100 sterilization wrap, O&M Halyard, Inc., Alpharetta, Georgia) is secured to the face shield and then draped over the patients head to create a closed environment.

We need to critically examine the effectiveness of the NPFS, on reducing virus aerosols to HCWs during patient care. The coughing machine is designed to simulate a coughing patient during a laryngoscopy procedure. The simulated coughing patient was outfitted with the two face shields and the virus and particle concentrations will be measured on the inside and outside of the face shields. This approach will allow the comparison of virus concentrations observed in the HCWs breathing zone between the two face shield designs. To meet this aim, we:

1. Analyzed viable virus concentrations and particle concentrations across a disposable plastic face shield that is placed on a simulated patient.
2. Analyzed viable virus concentrations and particle concentrations across the NPFS that is placed on a simulated patient.
3. Compared the viable virus concentrations and particle concentrations across the disposable plastic face shield and the NPFS.

## Materials and methods

### Power analysis

A power analysis was conducted a priori to determine the sample size needed to result in 80% power to detect the true mean of the population, the recommended level, when using a two-tailed t-test with an alpha (α) set at the 0.05 R (R Core Team (2020) (R: A language and environment for statistical computing. R Foundation for Statistical Computing, Vienna, Austria)). The determined sample size was 16.7 for each group; therefore, 17 aerosolization trials were targeted for each face shield experiment.

### Coughing machine virus aerosolization

A coughing machine was designed to simulate a patient that is coughing viral aerosol particles (article currently under review). In summary, the coughing machine aerosolized MS2, a surrogate virus for SARS-CoV-2. The MS2 was suspended in liquid broth (Appendix A) and aerosolized using the coughing machine. The coughing machine “exhales” a burst of air like a human cough. First, a disposable plastic face shield was placed on the simulated patient while the coughing machine aerosolized the virus surrogate. Then, the negative pressure face shield (NPFS)^11^. The NPFS was connected to a pump that is evacuating the air away from the patients face. Two flow rates of the face shield were tested, 10 LPM and 38.6 LPM. The 38.6 LPM was selected since it is the average flow rate used during patient care and 10 LPM was selected as a potential lower flow rate.

### Virus and particle concentrations

**Figure 1:**
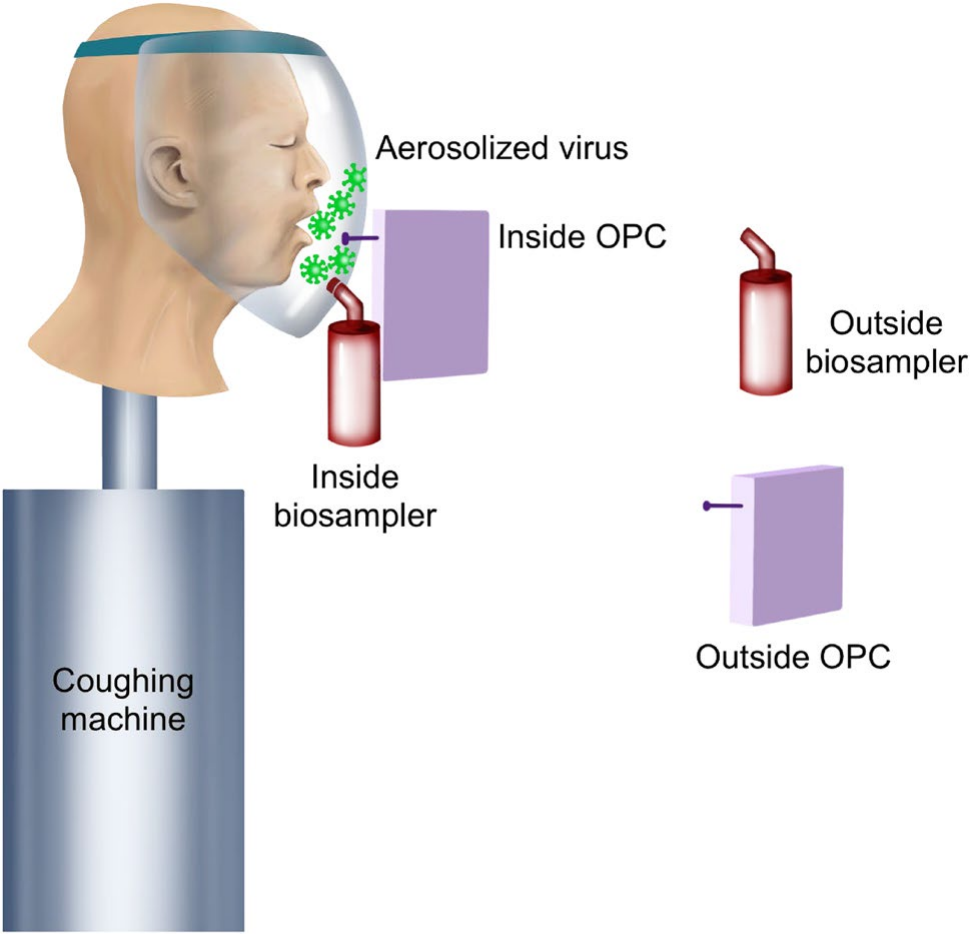
Schematic showing sampling setup when the disposable plastic face shield is placed on the simulated coughing patient. The face shield has two holes on the front for scope access to either the nose or mouth. The Biosamplers and OPCs are placed on the inside and outside of the face shield.

**Figure 2:**
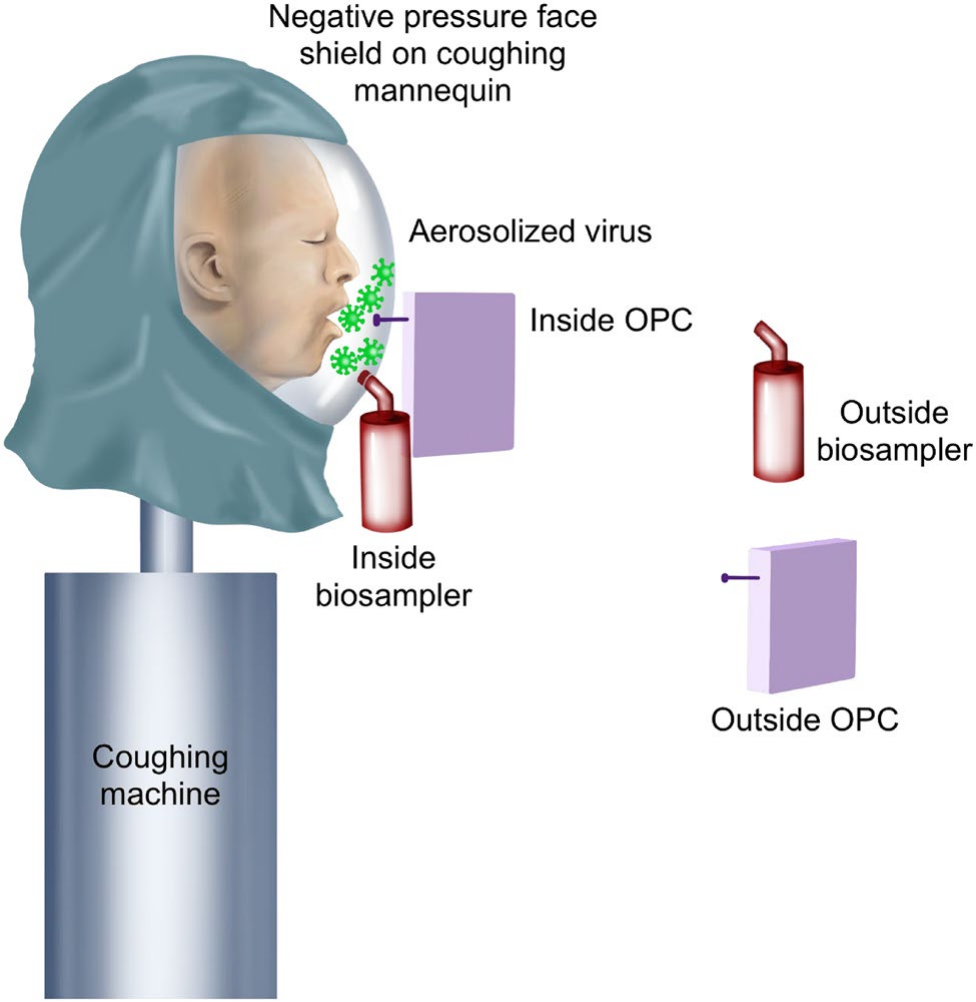
Schematic showing sampling setup when the negative pressure face shield is placed on the simulated coughing patient. The face shield has two holes on the front for scope entry into either the nose or mouth and a side port connecting the face shield to a pump evacuating air away from the patient. For the sampling holes were drilled in the front and bottom so the SKC Biosampler and OPC GRIMM can access the cough behind the face shield, near the oral cavity. The NPFS is connected to a pump evacuating air away from the coughing patient’s face at either 10 LPM or 38.6 LPM. The Biosamplers and OPCs are placed on the inside and outside of the face shield.

Virus and particle concentrations were sampled on the inside and outside of the two face shields placed on the simulated coughing patients. SKC Biosamplers (Biosamplers) (SKC Biosamplers, 225-9595, SKC Inc., Eighty-Four, PA, US) were used to sample the MS2 (virus concentration) and GRIMM 11D Optical Particle Counters (OPCs) (GRIMM Aerosol Counters, Durag Group, Germany, SN# 619012 and 619025) were used to sample the particle concentration. The inlets of the Biosamplers and OPCs were placed on the inside and outside of the patient’s disposable plastic face shield (Fig. 1) and the NPFS (Fig. 2). The liquid media used in the Biosampler was 20 mL of phosphate buffered saline (PBS) (Gibco, 1x, pH 7.4 -/-, Texas, USA). The Biosamplers were each connected to a pump that pulled air through the samplers at 12.5 LPM. The outlet of the pump was connected to a HEPA filter. The OPCs, with a flow rate of 1.2 LPM, sampled at one second across sixteen particle (bin) sizes (ranging from 0.253 to 3.515 µm) reporting particle number concentration data. The Biosamplers and OPCs were started one minute prior to the first cough and operated nonstop throughout the three coughs (approximately 28 s). After the coughs were completed, the Biosamplers and OPCs were turned off and the sample were analyzed.

The sampling was conducted in a chamber-like room at a remote laboratory building (8.5 m (m) × 3.7 m × 2.4 m). All ventilation to the room was turned off, there was limited traffic, and doors and windows were shut. This approach allowed the sampling room to be used as an undisturbed chamber. The room also maintained a consistent relative humidity and temperature and was measured using a direct reading instrument (Extech Instruments Hygro-Thermometer Model SDL500, SN: Z335535) throughout all trials. Post sampling, the chamber-like room, and the coughing machine were cleaned with sodium hypochlorite wipes and 70% ethanol.

### Virus sample analysis

A plaque assay was used to analyze the concentration and viability of the Biosampler liquid media. The sample, 20 mL of PBS that was run through the Biosampler, was be concentrated using an Amicon Ultra 15 Centrifugal Filters (nominal molecular weight limit 50 KDa, Cork, Ireland) which is spun in the centrifuge at 4000 rotations per minute (RPM) in one-minute intervals until the remaining sample volume is less than 1000 µL. The concentrated sample volume was measured and recorded. A serial dilution was then conducted using 100 µL of the concentrated sample and 900 µL of MS2 broth (Appendix A). Petri dishes (Fisher Scientific, 100 mm × 15 mm, Polystyrene, Massachusetts, USA) for the plaque assay were prepared using 10 mL of bottom agar which was left to rest for 30 min before any overlay is added. Top agar was prepared and aliquoted into 3 mL tubes (FalconBrand, 14 mL polystyrene round bottom tubes, Mexico) and incubated for 20 min at 45 °C. After the incubation period, 10 µL of liquid *E. coli* (10^7^ PFU/mL) (ATCC15597) and 100 µL of the serial dilution sample was added to the 3 mL of top agar, this was then mixed and poured over the solidified bottom agar plates. Each serial dilution was plated in triplicate. The plates were left to solidify for 20 min and then sealed with a thermoplastic film (Parafilm wrapping film, Bemis, RPI, Wisconsin, USA). The sealed plates were placed upside down in a 37 °C incubator for 14–24 h. After the incubation period, the MS2 plaques formed. The serial dilution plate that produced between 20 and 250 plaques were then counted. The average across the triplicate measure was taken. Blank samples were used throughout the sampling chamber and ventilation hood to ensure no contamination.

The plaque count and dilution factor were used to calculate the plaque concentration (Eq. 1). The plaque concentration, concentrated volume, and air volume sampled were then used to determine the airborne concentration of MS2 (Eq. 2).

Plaque Concentration Formula

1 $$\mathrm{Plaque\;Concentration }\left(\mathrm{PFU}\right)=\left(\mathrm{Average \; plaque \; forming \; units}\right)/(\mathrm{Total \;volume \;plated})$$

Sample Concentration Formula

2 $$\mathrm{Sample \;Concentration }\left(\mathrm{PFU}/{\mathrm{m}}^{3}\right)=\mathrm{ Plaque \; concentration }(\mathrm{PFU})/\mathrm{Volume\; of\; air \;sampled }({\mathrm{m}}^{3})$$

The normality of the three separate face shield conditions was analyzed using Shapiro-Wilks test. The three conditions each had different sample sizes: disposable plastic face shield (n = 17), NPFS at “low flow rate” or 10 LPM (n = 6), and NPFS at a “high flow rate” or 38.6 LPM (n = 18). The censored data for the virus concentration was replaced with the LOD/2. Arithmetic means and standard deviations were computed across the trials for the inside and outside of the face shield. A paired t-test was performed to compare the two groups.

### Particle sample analysis

The OPCs were connected to two computers which each ran the OPC Software (Spectrometer, GRIMM Control, 1179, V1-0-6, 25-10-2018). The particle data were exported to Microsoft Excel (Microsoft Office Professional Plus 2016, V16.0.5278.1000) for analysis. The inside and outside face shield particle data for all the trials in one condition was compiled for each particle size. The aerosol particle data were determined to be log-normally distributed and were log-transformed. The variance was tested and based off the results, an appropriate t-test was selected and then conducted for comparing the inside and outside of the face shield for each particle size. The paired t-test was conducted using an unadjusted alpha of 0.05 as well as an alpha that was adjusted for False Discovery Rate using the Benjamini Hochberg procedure^12^. Descriptive statistics were calculated, specifically the geometric mean and geometric standard deviation. A box plot showing the median first quartile, third quartile, standard deviations and outliers was also made for each condition. For each variable, outliers were identified as values greater than 1.5 interquartile range (IQR) third quartile or 1.5 IQR lesser than the first quartile.

## Results

### Virus concentration

The starting concentration of the MS2 was 1 × 10E+09 PFU/m^3^. This concentration was verified after the aerosolization-coughing event ensure consistency within each trial. The airborne concentration inside the face shield, near the breathing zone of the patient, when the patient was wearing the disposable plastic face shield had a mean (n = 16) of 1.27E+09 PFU/m^3^ (Standard Deviation (SD) = 8.15E+08) (Table 1). The calculated airborne concentration outside the face shield, near the breathing zone of the HCW, when the patient was wearing the typical disposable plastic face shield had a mean (n = 16) of 1.24E+08 PFU/m^3^ (SD = 9.11E+07) (Table 1). The calculated airborne concentration inside the face shield, near the breathing zone of the patient, when the patient was wearing the NPFS that was operated at the high flow rate had a mean (n = 18) of 1.20E+06 PFU/m^3^ (SD = 8.17E+05) (Table 1). The calculated airborne concentration outside the face shield, near the breathing zone of the HCW, when the patient was wearing NPFS that was operated at the high flow rate was unable to be detected (Table 1). The calculated airborne concentration inside the face shield, near the breathing zone of the patient, when the patient was wearing the NPFS that was operated at the low flow rate had a mean (n = 6) of 5.74E+08 PFU/m^3^ (SD = 6.44 E+08) (Table 1). The calculated airborne concentration outside the face shield, near the breathing zone of the HCW, when the patient was wearing NPFS that was operated at the high flow rate was 1.73E+08 PFU/m^3^ (SD = 2.57E+08) (Table 1).

**Table 1:** Mean (SD) Concentrations of viable MS2 virus inside and outside the three face shields of the simulated patient during a simulated coughing event.

Face shield type	Sample size, N	MS2 mean concentrations (SD) (PFU/m^3^)		p-value
		Inside	Outside	
Disposable plastic	17	1.27E+09 (8.15E+08)	1.24E+08 (9.11E+07)	0.12
NPFS, 38.6 LPM	18	1.20E+06 (8.17E+05)	ND (ND, ND)	* < 0.001
NPFS, 10 LPM	5	5.74E+08 (6.44E+08)	1.73E+08 (2.57E+08)	0.09

Significant values are in bold.

### Particle concentration

**Figure 3:**
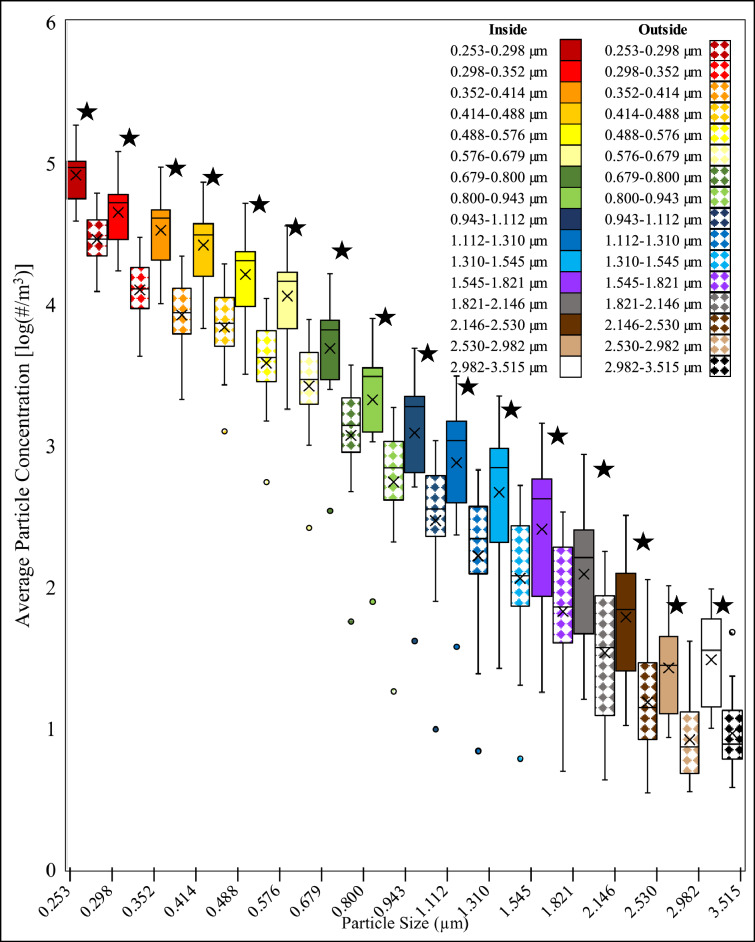
Box plot average particle concentration comparing the inside and outside of the modified disposable plastic face shield placed on a coughing patient. The inside of the face shield is solid filled and the outside of the shield is pattern filled. The data is organized by increasing particle size. Statistically significant pairs are marked with stars.

The variance was tested and determined to be unequal so a t-test assuming unequal variance was conducted for comparing the inside and outside of the face shield for each particle size. The box plot for the number particle concentration on the inside and outside of the typical disposable plastic face shield, worn by the patient, across all particle sizes is shown in (Fig. 3). The paired t-test for comparing the particle concentrations for each particle size on the inside and outside of the face shield is shown in Appendix B. Findings from the paired t-test showed all the particle sizes had a statistically significant decrease in number concentration between the inside and outside of the face shield (P < 0.05).

**Figure 4:**
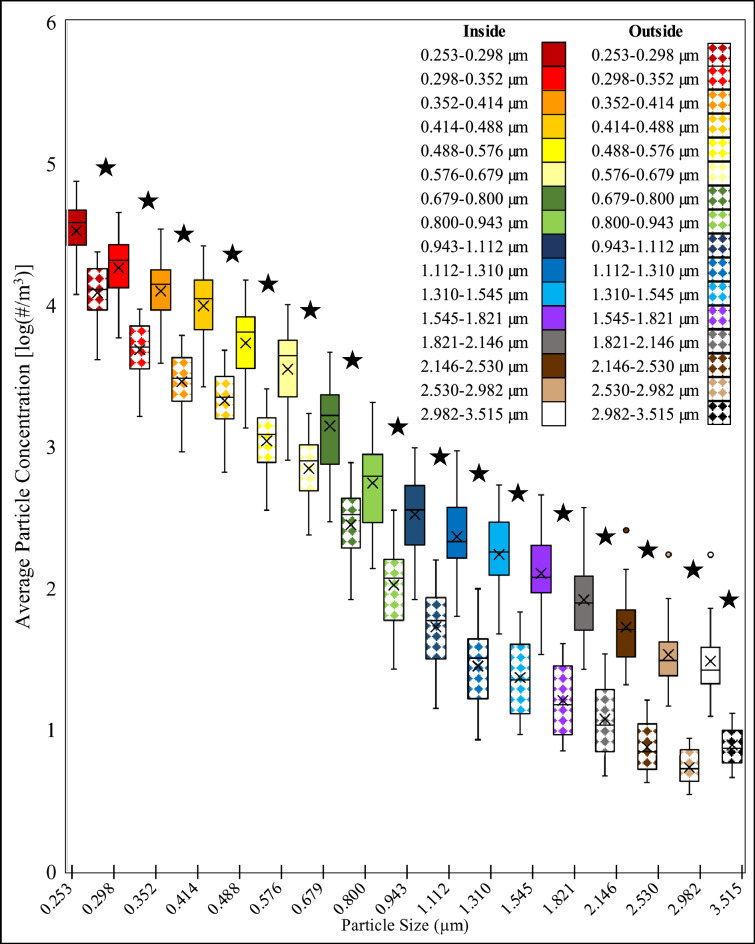
Box plot of the average particle concentration when High Flow (flow rate is ~ 38.6 LPM) Negative Pressure Face Shield is placed on coughing patient comparing the inside and outside. The inside of the face shield is solid filled and the outside of the shield is pattern filled. The data is organized by increasing particle size. Statistically significant pairs are marked with stars.

All the particle sizes were significantly lower in number concentration between the inside and outside of the face shield when the NPFS that was operated at a high flow rate (Fig. 4, Appendix C).

**Figure 5:**
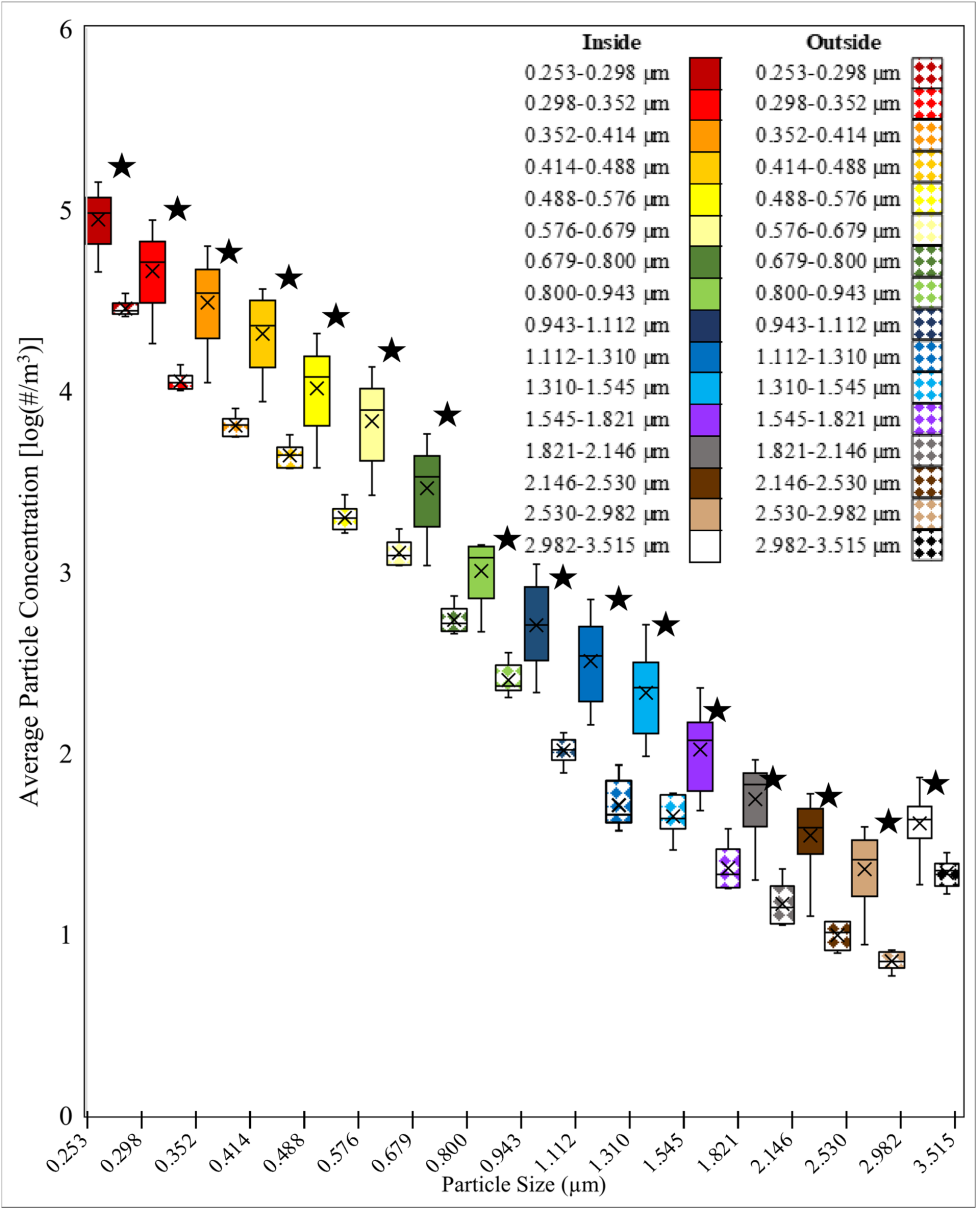
Box plot of the average particle concentration when Low Flow (flow rate is 10 LPM) Negative Pressure Face Shield is placed on coughing patient comparing the inside and outside. The inside of the face shield is solid filled and the outside of the shield is pattern filled. The data is organized by increasing particle size. Statistically significant pairs are marked with stars.

The box plot for the number particle concentration on the inside and outside of the NPFS that is operated at a low flow rate, across all particle sizes is shown in (Fig. 5). The paired t-test for comparing the particle concentrations for each particle size on the inside and outside of the face shield is shown in Appendix D. Findings from the paired t-test showed all the particle sizes had a significant difference, using the FDR adjusted P-Value, in number concentration between the inside and outside of the face shield.

## Discussion

The goal of this experiment was to analyze the effectiveness of the NPFS at controlling spread virus-laden bioaerosol during simulated coughing. The NPFS could be an effective approach to decrease room contamination and HCW exposure during aerosol generating procedures (e.g., laryngoscopy). We analyzed viable virus and particle concentrations across a disposable plastic face shield modified for laryngoscopy procedures, a high flow NPFS and a low flow NPFS placed on a patient during a simulated cough. The measurements taken on the inside of the face shield were used to see if there was any reduction in MS2, the surrogate for SARS-CoV-2, concentration exposure to the outside of the face shield where the HCWs would be exposed. To see if there was a difference between the inside and outside of the face shields, we performed statistical analysis to compare the virus and particle concentrations found across the face shield. The goal of this experiment was to analyze the effectiveness of the face shield at reducing virus exposure so that we can improve control measures for protecting HCWs during high-risk procedures.

The NPFS is a novel control device identified as effective in permitting effective transnasal laryngoscopy for diagnosis and procedures with excellent patient tolerance^13^. To date the effectiveness in controlling bioaerosol had not been tested. However, combining PPE and engineering controls is more effective than PPE alone^7^. In addition, studies have shown that PPE needs to be updated and engineered to meet the needs of HCWs and the NPFS does this^14^.

When the simulated coughing patient was wearing a disposable plastic face shield during a coughing event, we did not observe a statistically significant difference in airborne virus concentration between the inside and outside of the face shield (p = 0.12). We did observe a statistically significant decrease (p < 0.05) in particle number concentrations between the inside and outside of the face shield for all particle sizes measured.

When the NPFS was operated at a high flow during a coughing event, we did observe a statistically significant decrease in airborne virus concentration outside of the face shield (p < 0.05). Specifically, no virus was measured on the outside of the NPFS, where the HCW would be exposed. We also observed a statistically significant decrease (p < 0.05) in particle number concentrations outside of the face shield for all particle sizes measured. The inner quartile ranges and number of outliers were reduced when operating the NPFS’s compared to the disposable plastic face shield.

When the NPFS operated at a low flow during a coughing event, we did not observe a statistically significant difference in airborne virus concentration between the inside and outside of the face shield (p = 0.09). We did observe a statistically significant difference (p < 0.05) in particle number concentrations between the inside and outside of the face shield for all particle sizes.

The virus concentration was significantly reduced for the high flow NPFS and was reported at 0. We believe that our sampled amount was in fact zero because the particle concentration did not increase on the outside of the face shield when the coughing trials were run. Our coughing machine may have affected virus viability, which would in turn impact the concentrations of the plaque assay. The virus concentrations on the inside and outside of the typical disposable plastic face shield and the low flow NPFS were not significantly different. This means that the HCW is still potentially exposed to viable virus aerosols when those control methods are used. However, while not significantly reduced, it is important to note that any reduction in concentration reduces exposure of virus to the HCW and is therefore important. While the reduction may not eliminate the risk of transmission it may reduce risk below the threshold needed for infection. Further work should be done to quantify the concentration of airborne virus needed for infection. There were a few notable limitations in this study. The experiment was performed in a chamber like room with no room ventilation. In health care environments, ventilation is present which may result in different observations. Specifically, in the otolaryngology surgical clinic, rooms may be under negative pressure, which may allow for additional protection for the HCW.

We did not correct for background aerosol concentration in any of these experiments. Our experimental approach was designed using paired comparisons (i.e., paired t-test) so this limitation should have little if any impact on our observations of face shield effectiveness. The background concentrations should be the same for the inside and outside of the two face shields including the NPFS since it was pulling air from just outside the face shield. Further, we measured live virus concentrations across the face shield rather than solely relying on airborne particle concentrations and the results from the live virus and particle concentrations were consistent across all face shield scenarios further validating this decision. The virus aerosol concentration aerosolized may have varied across trials. To control for this error, we measured virus plaque concentrations in the stock solution before each experiment. Furthermore, our comparisons and statistical analysis was paired which would reduce the impact of this error on our experiment. In addition, there may have been variance in our virus concentration estimates due to using a plaque assay as our method of quantification. To mitigate this limitation, we used a triplicate measure when performing the plaque assay and took the numerical mean. In the future a more sensitive analysis, like digital droplet polymerase chain reaction (ddPCR) could be utilized. We chose not to utilize ddPCR because we were interested only in the viable virus concentration in order to analyze infectious particles a HCW would be exposed to. However, ddPCR could be very informative to negate the limitation that the coughing machine was inactivating the virus which would make the plaque assay not a viable analysis method.

The particle concentrations were significantly different for all three tested face shields. This implies that the new design of face shields reduced some aerosol from the coughing plume. Some studies have found that face shields can reduce exposure^15^. However, most studies have analyzed how effective face shields are at protecting HCWs from infectious patients when the HCW wears the face shield, making our work novel^16^. In addition, by using the GRIMM 11-D OPC we introduce an inherent limitation in that the smallest bin size that it can accurately detect is 0.253 µm. We hope that this limitation is mitigated by the MS2 being laden in liquid media and therefore creating a droplet during the coughing event however, there is still a limitation that the smallest airborne virus particles were not analyzed. We also further tried to validate our approach by analyzing virus concentration in addition to particle concentration. While we chose the OPC for its sensitivity, flow rate, and detection limit further work should be done which analyzes smaller particle sizes.

The sample sizes across the three different face shield scenarios varied. The lowest sample size was for the low flow NPFS with a total sample size of six. We chose not to continue to pursue the lower flow rate and meet the sample size needed to reach a power of 0.8 because the there was still viable virus being measured on the outside of the face shield. We chose to focus on the higher flow rate and the disposable plastic face shield since that flow rate has been used in clinical practice.

The mannequin head of the coughing machine is not the same size as an average head so the typical disposable plastic face shield did not fit the same as it would. Since the head was smaller, the face shield wrapped further around the head and our results were probably overestimating the effectiveness of the control measure. In conclusion, we recommend that the high flow NPFS be considered for use during higher-risk aerosol generating endoscopy procedures to limit the potential transmission of virus to HCWs. We recommend that future studies be conducted with the NPFS on patients because the added effect of aerosol generation from the procedures was not analyzed, only the aerosol generated from the patient coughing.

### Supplementary Information

Supplementary Information.

## Data Availability

The datasets generated during and/or analyzed during the current study are available from the corresponding author on reasonable request.
