# Supplementary material for: Evaluation of face shields used during aerosol generating procedures
Source: Sci Rep. 2023 Sep 20;13:15617. doi: 10.1038/s41598-023-42403-8 (PMC10511466; doi:10.1038/s41598-023-42403-8)
Supplement: Supplementary file 1 — Supplementary Information. [file 41598_2023_42403_MOESM1_ESM.docx]

Appendix A

MS2 Broth Solution Recipe

| Ingredient | Broth | Bottom Agar | Top Agar |
| --- | --- | --- | --- |
| Tryptone (g) | 5 | 5 | 5 |
| Yeast Extract (g) | 0.5 | 0.5 | 0.5 |
| NaCl (g) | 4 | 4 | 4 |
| Agar (g) | 0 | 7.5 | 2.5 |
| Supplement (mL) | 25 | 25 | 25 |
| DI Water (mL) | 475 | 475 | 475 |

Makes 500mL. All solutions autoclaved without added supplement at 121°C for 20 minutes then placed in a water bath (55°C) to cool. The supplement was added and mixed once the solution cooled

Solution Recipe of supplement

| Ingredient | Supplement |
| --- | --- |
| Glucose (g) | 10 |
| CaCl_2_ (g) | 2.934 |
| Thiamine [17] | 100 |
| DI Water (mL) | 500 |

Makes 500mL. Filter solution with 0.22µm pore filter prior to use.

Appendix B: Paired t-test results for comparing the inside and outside of the disposable face shield across particle size when the face shield is placed on the simulated coughing patient.

| Particle Size (µm) | Location | Mean  (log(#/m^3^)) | Variance  ((log(#/m^3^))^2^) | df | t Stat | Unadjusted P-Value | FDR Adjusted P-Value |
| --- | --- | --- | --- | --- | --- | --- | --- |
| 0.253-0.298 | Inside | 4.913 | 0.029 | 17 | 15.115 | <0.01* | <0.01* |
|  | Outside | 4.460 | 0.030 |  |  |  |  |
| 0.298-0.352 | Inside | 4.650 | 0.043 | 17 | 15.071 | <0.01* | <0.01* |
|  | Outside | 4.101 | 0.043 |  |  |  |  |
| 0.352-0.414 | Inside | 4.524 | 0.055 | 17 | 15.045 | <0.01* | <0.01* |
|  | Outside | 3.923 | 0.059 |  |  |  |  |
| 0.414-0.488 | Inside | 4.419 | 0.059 | 17 | 15.093 | <0.01* | <0.01* |
|  | Outside | 3.837 | 0.076 |  |  |  |  |
| 0.488-0.576 | Inside | 4.209 | 0.076 | 17 | 14.893 | <0.01* | <0.01* |
|  | Outside | 3.584 | 0.089 |  |  |  |  |
| 0.576-0.679 | Inside | 4.058 | 0.085 | 17 | 14.958 | <0.01* | <0.01* |
|  | Outside | 3.419 | 0.110 |  |  |  |  |
| 0.679-0.800 | Inside | 3.687 | 0.132 | 17 | 14.535 | <0.01* | <0.01* |
|  | Outside | 3.071 | 0.157 |  |  |  |  |
| 0.800-0.943 | Inside | 3.323 | 0.185 | 17 | 13.413 | <0.01* | <0.01* |
|  | Outside | 2.741 | 0.196 |  |  |  |  |
| 0.943-1.112 | Inside | 3.088 | 0.211 | 17 | 14.163 | <0.01* | <0.01* |
|  | Outside | 2.469 | 0.216 |  |  |  |  |
| 1.112-1.310 | Inside | 2.877 | 0.210 | 17 | 13.414 | <0.01* | <0.01* |
|  | Outside | 2.218 | 0.242 |  |  |  |  |
| 1.310-1.545 | Inside | 2.668 | 0.223 | 17 | 10.444 | <0.01* | <0.01* |
|  | Outside | 2.060 | 0.226 |  |  |  |  |
| 1.545-1.821 | Inside | 2.408 | 0.256 | 17 | 10.646 | <0.01* | <0.01* |
|  | Outside | 1.824 | 0.249 |  |  |  |  |
| 1.821-2.146 | Inside | 2.089 | 0.210 | 17 | 8.646 | <0.01* | <0.01* |
|  | Outside | 1.532 | 0.218 |  |  |  |  |
| 2.146-2.530 | Inside | 1.785 | 0.155 | 17 | 10.973 | <0.01* | <0.01* |
|  | Outside | 1.183 | 0.149 |  |  |  |  |
| 2.530-2.982 | Inside | 1.425 | 0.099 | 17 | 9.619 | <0.01* | <0.01* |
|  | Outside | 0.916 | 0.083 |  |  |  |  |
| 2.982-3.515 | Inside | 1.484 | 0.095 | 17 | 10.297 | <0.01* | <0.01* |
|  | Outside | 0.961 | 0.073 |  |  |  |  |

* Statistically significant (<0.05) P-Value, df= degrees of freedom

Appendix C: Paired t-test results for high flow negative pressure face shield comparing the inside and outside of the face shield across particle size.

| Particle Size (µm) | Location | Mean  (log(#/m^3^)) | Variance  ((log(#/m^3^))^2^) | df | t Stat | Unadjusted P-Value | FDR Adjusted P-Value |
| --- | --- | --- | --- | --- | --- | --- | --- |
| 0.253-0.298 | Inside | 4.524 | 0.052 | 17 | 11.539 | <0.01* | <0.01* |
|  | Outside | 4.086 | 0.044 |  |  |  |  |
| 0.298-0.352 | Inside | 4.261 | 0.062 | 17 | 13.535 | <0.01* | <0.01* |
|  | Outside | 3.683 | 0.046 |  |  |  |  |
| 0.352-0.414 | Inside | 4.098 | 0.069 | 17 | 13.858 | <0.01* | <0.01* |
|  | Outside | 3.458 | 0.050 |  |  |  |  |
| 0.414-0.488 | Inside | 3.993 | 0.076 | 17 | 14.424 | <0.01* | <0.01* |
|  | Outside | 3.322 | 0.051 |  |  |  |  |
| 0.488-0.576 | Inside | 3.730 | 0.087 | 17 | 14.247 | <0.01* | <0.01* |
|  | Outside | 3.038 | 0.052 |  |  |  |  |
| 0.576-0.679 | Inside | 3.545 | 0.096 | 17 | 13.149 | <0.01* | <0.01* |
|  | Outside | 2.843 | 0.051 |  |  |  |  |
| 0.679-0.800 | Inside | 3.143 | 0.112 | 17 | 12.476 | <0.01* | <0.01* |
|  | Outside | 2.446 | 0.070 |  |  |  |  |
| 0.800-0.943 | Inside | 2.741 | 0.096 | 17 | 12.007 | <0.01* | <0.01* |
|  | Outside | 2.020 | 0.089 |  |  |  |  |
| 0.943-1.112 | Inside | 2.519 | 0.089 | 17 | 13.583 | <0.01* | <0.01* |
|  | Outside | 1.724 | 0.084 |  |  |  |  |
| 1.112-1.310 | Inside | 2.362 | 0.086 | 17 | 13.480 | <0.01* | <0.01* |
|  | Outside | 1.448 | 0.082 |  |  |  |  |
| 1.310-1.545 | Inside | 2.236 | 0.082 | 17 | 13.740 | <0.01* | <0.01* |
|  | Outside | 1.366 | 0.072 |  |  |  |  |
| 1.545-1.821 | Inside | 2.102 | 0.082 | 17 | 14.518 | <0.01* | <0.01* |
|  | Outside | 1.203 | 0.063 |  |  |  |  |
| 1.821-2.146 | Inside | 1.914 | 0.081 | 17 | 13.071 | <0.01* | <0.01* |
|  | Outside | 1.073 | 0.067 |  |  |  |  |
| 2.146-2.530 | Inside | 1.722 | 0.068 | 17 | 15.123 | <0.01* | <0.01* |
|  | Outside | 0.874 | 0.035 |  |  |  |  |
| 2.530-2.982 | Inside | 1.528 | 0.063 | 17 | 14.648 | <0.01* | <0.01* |
|  | Outside | 0.731 | 0.016 |  |  |  |  |
| 2.982-3.515 | Inside | 1.481 | 0.075 | 17 | 10.572 | <0.01* | <0.01* |
|  | Outside | 0.889 | 0.017 |  |  |  |  |

* Statistically significant (<0.05) P-Value, df= degrees of freedom

Appendix D: Paired t-test results for low flow negative pressure face shield comparing the inside and outside of the face shield across particle size.

| Particle Size (µm) | Location | Mean  (log(#/m^3^)) | Variance  ((log(#/m^3^))^2^) | df | t Stat | Unadjusted P-Value | FDR Adjusted P-Value |
| --- | --- | --- | --- | --- | --- | --- | --- |
| 0.253-0.298 | Inside | 4.943 | 0.029 | 5 | 7.549 | <0.01* | <0.01* |
|  | Outside | 4.452 | 0.002 |  |  |  |  |
| 0.298-0.352 | Inside | 4.659 | 0.054 | 5 | 6.921 | <0.01* | <0.01* |
|  | Outside | 4.049 | 0.003 |  |  |  |  |
| 0.352-0.414 | Inside | 4.484 | 0.067 | 5 | 7.182 | <0.01* | <0.01* |
|  | Outside | 3.805 | 0.003 |  |  |  |  |
| 0.414-0.488 | Inside | 4.312 | 0.050 | 5 | 8.364 | <0.01* | <0.01* |
|  | Outside | 3.640 | 0.005 |  |  |  |  |
| 0.488-0.576 | Inside | 4.010 | 0.066 | 5 | 7.704 | <0.01* | <0.01* |
|  | Outside | 3.297 | 0.005 |  |  |  |  |
| 0.576-0.679 | Inside | 3.830 | 0.062 | 5 | 7.711 | <0.01* | <0.01* |
|  | Outside | 3.103 | 0.006 |  |  |  |  |
| 0.679-0.800 | Inside | 3.459 | 0.064 | 5 | 7.460 | <0.01* | <0.01* |
|  | Outside | 2.732 | 0.006 |  |  |  |  |
| 0.800-0.943 | Inside | 3.002 | 0.034 | 5 | 8.546 | <0.01* | <0.01* |
|  | Outside | 2.401 | 0.008 |  |  |  |  |
| 0.943-1.112 | Inside | 2.703 | 0.062 | 5 | 6.253 | <0.01* | <0.01* |
|  | Outside | 2.011 | 0.006 |  |  |  |  |
| 1.112-1.310 | Inside | 2.506 | 0.060 | 5 | 7.762 | <0.01* | <0.01* |
|  | Outside | 1.710 | 0.019 |  |  |  |  |
| 1.310-1.545 | Inside | 2.329 | 0.062 | 5 | 7.585 | <0.01* | <0.01* |
|  | Outside | 1.648 | 0.013 |  |  |  |  |
| 1.545-1.821 | Inside | 2.016 | 0.056 | 5 | 6.974 | <0.01* | <0.01* |
|  | Outside | 1.361 | 0.016 |  |  |  |  |
| 1.821-2.146 | Inside | 1.742 | 0.055 | 5 | 5.767 | <0.01* | <0.01* |
|  | Outside | 1.164 | 0.014 |  |  |  |  |
| 2.146-2.530 | Inside | 1.542 | 0.054 | 5 | 5.599 | <0.01* | <0.01* |
|  | Outside | 0.992 | 0.007 |  |  |  |  |
| 2.530-2.982 | Inside | 1.356 | 0.051 | 5 | 7.460 | <0.01* | <0.01* |
|  | Outside | 0.848 | 0.003 |  |  |  |  |
| 2.982-3.515 | Inside | 1.609 | 0.036 | 5 | 4.303 | <0.01* | <0.01* |
|  | Outside | 1.334 | 0.006 |  |  |  |  |

* Statistically significant (<0.05) P-Value, df= degrees of freedom
